# Supplementary material for: Polycotton waste textile recycling by sequential hydrolysis and glycolysis
Source: Nat Commun. 2025 Jan 29;16:738. doi: 10.1038/s41467-025-55935-6 (PMC11779930; doi:10.1038/s41467-025-55935-6)
Supplement: Supplementary file 1 — Supplementary Information [file 41467_2025_55935_MOESM1_ESM.pdf]

Supplementary Information

Polycotton waste textile recycling by sequential hydrolysis and glycolysis

Nienke Leenders [1], Rijk M. Moerbeek [2], Matthijs J. Puijk [3], Robbert J. A. Bronkhorst [3], Jorge Bueno Morón [3], Gerard P. M. van Klink [1,3] & Gert-Jan M. Gruter [1,3] *

Affiliations

[1] *Van 't Hoff Institute for Molecular Sciences, University of Amsterdam, Science Park 904, 1090 GD Amsterdam (the Netherlands)*

[2] *Faculty of Science and Technology, Hogeschool Leiden, Zernikedreef 11, 2333 CK Leiden (the Netherlands)*

[3] *Avantium Support BV, Zekeringstraat 29, 1014 BV Amsterdam (the Netherlands)*

*Corresponding author. E-mail address: g.j.m.gruter@uva.nl

Table S1: Overview of larger scale chemical recycling processes focusing on cotton recycling. Adapted from Loo et al. (2023).[1]

Company	Feedstock	Method	Product	Challenges	Scale	Reference
BlockTexx	Polycotton waste	Acid hydrolysis with H_2SO_4 at high temperature and pressure (120–150 °C at 5 bar)	Cellulose clay and PET pellets	High CAPEX cost due to the use H_2SO_4 at high pressure and temperature	Have a 10 kt pilot plant in Queensland, AU	(2)
Circ	Polycotton waste	Hydrothermal treatment with subcritical water	Lyocell and recycled polyester fibres	Extremely energy intensive	Are setting up a pilot plant, should be operational in 2024	(3)
EVRNU	Cotton rich waste	Dissolution in ionic liquids, organic solvents, metal alkali system complexes or other solvents	NuCycl lyocell fibre	Requires extensive pretreatment (Aqueous washing (>100 °C, pressurized), Supercritical CO_2 washing, Ozone enrichment)	Currently building first pilot plant (17 kt/a) in South Carolina, US	(4)
HKRITA	Polycotton waste	Hydrothermal treatment at 110–150 °C for 2 hours	Cellulose powder and PET residue	Extremely energy intensive	PT. Kahatex build a pilot plant (1.5 t/d) in Indonesia	(5)
Infinited Fiber	Cotton rich waste (>88% cotton)	Dissolution with alkali and urea treatment at elevated temperatures	Infinna (Cellulose carbamate)	Process requires a high cotton quantity	Currently building a flagship plant (30 kt/a) in Kemi, FI which will be operating in 2024	(6,7)
Ioncell	Polycotton waste	Dissolution in ionic liquid	Ioncell fibres and PET residue	Use of ionic liquid	Are currently looking for partners to go into pilot plant phase	(8)
Lenzing	Cotton waste	REFIBRA technology (dissolution in NMMO)	TENCEL Lyocell fibre	Can only process 100% cotton materials	Current Tencel production capacity is 140 kt/p.a	(9)
Renewcell	Cotton rich waste (>95% cotton)	Dissolution in NaOH	Circulose	Process requires a high cotton quantity	After building the pilot plant in 2017, they were deemed to file for bankruptcy in February 2024	(10)
SaXcell	Polycotton waste	Dissolution in NMMO at 85–100 °C	SaXcell and PET residue	Initial pilot plant scale only	Operating pilot plant (25 t/a) in Goor, NL	(11)
Sodra	White cotton rich waste (>70% cotton)	Dissolution in NaOH	OnceMore pulp	Strick feedstock requirements	Pilot plant (6 kt/a) in Mörrom, SE	(12,13)
Worn Again Technologies	Polycotton waste	Dissolution in ionic liquid	Cellulose pulp and polyester pellets	Use of ionic liquid	Demo plant (1 kt) in Winterthur, CH will be opened in 2024 after successful pilot plant (5 kg scale) in 2019	(14)

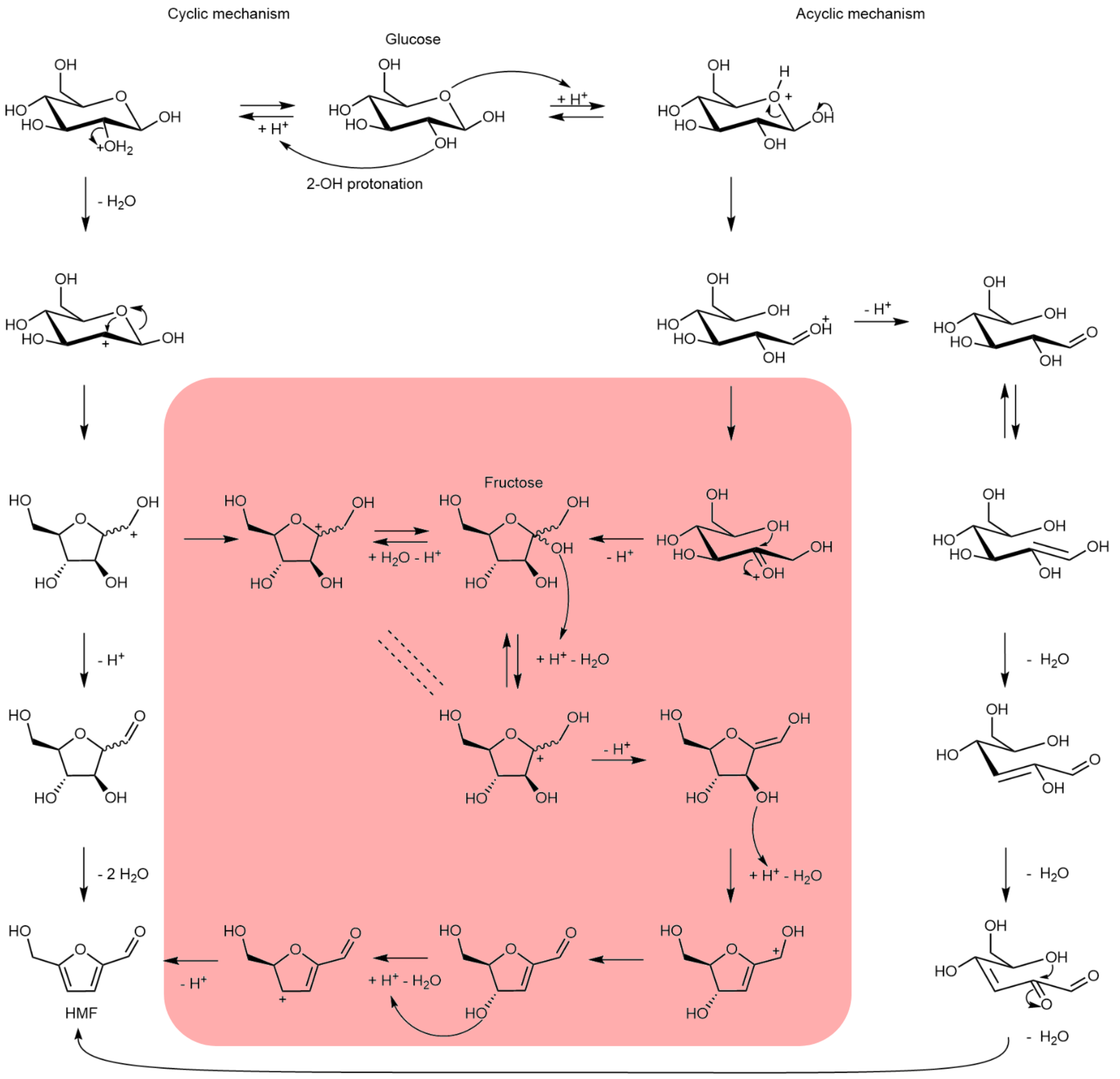

Figure S1: Proposed reaction mechanism for the dehydration of glucose to 5-(hydroxymethyl)furfural via cyclic and acyclic mechanisms.[15-17]

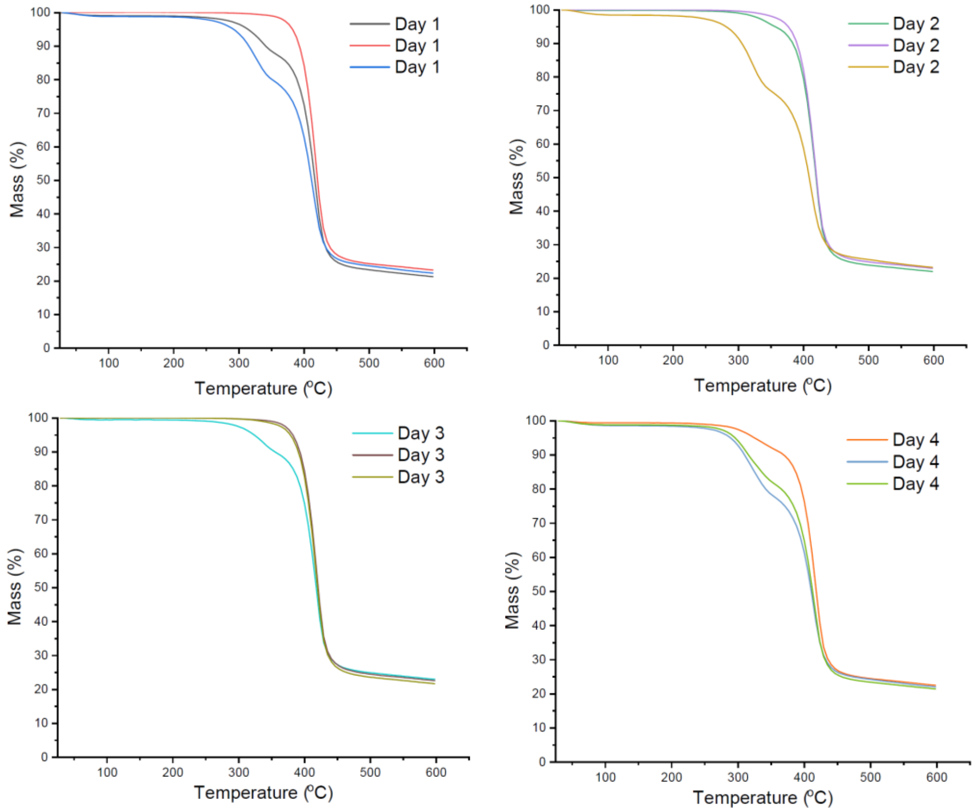

Figure S2: TGA of residual postconsumer waste after treatment with 37 wt% aq. HCl for 1–4 days measured under N_2 flow.

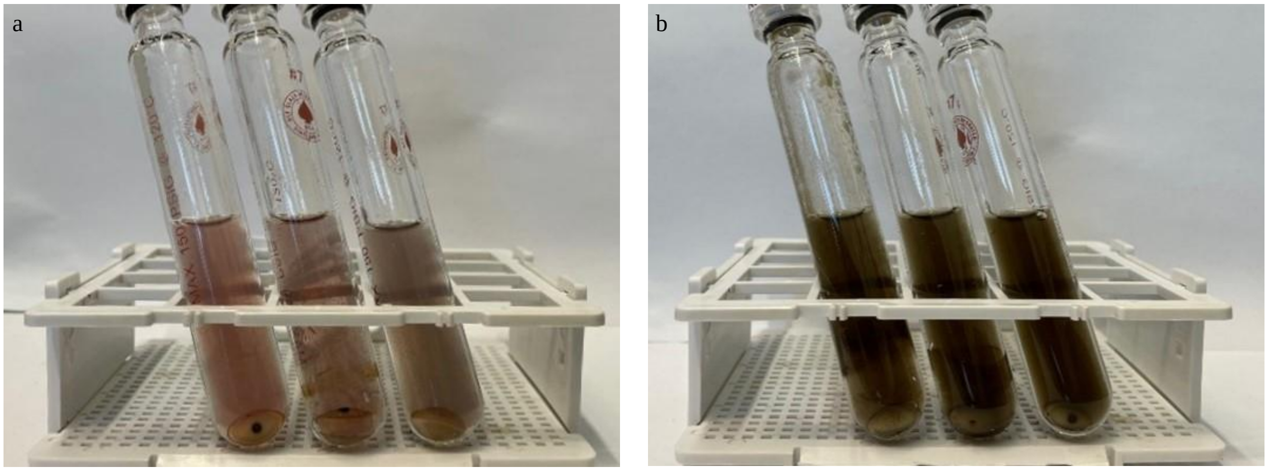

Figure S3: Images of the laboratory ACE glass reactors after acid hydrolysis with 37 wt% aq. HCl (a) and 43 wt% aq. HCl (b) after 4 days (50 mg/mL, 1000 rpm).

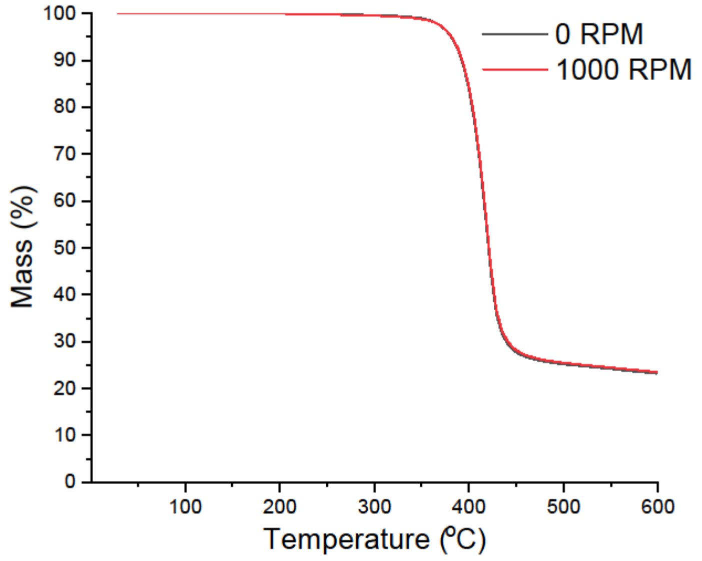

Figure S4: TGA of residual postconsumer waste textile after treatment with 43 wt% aq. HCl content at different stirring speeds measured under N_2 flow.

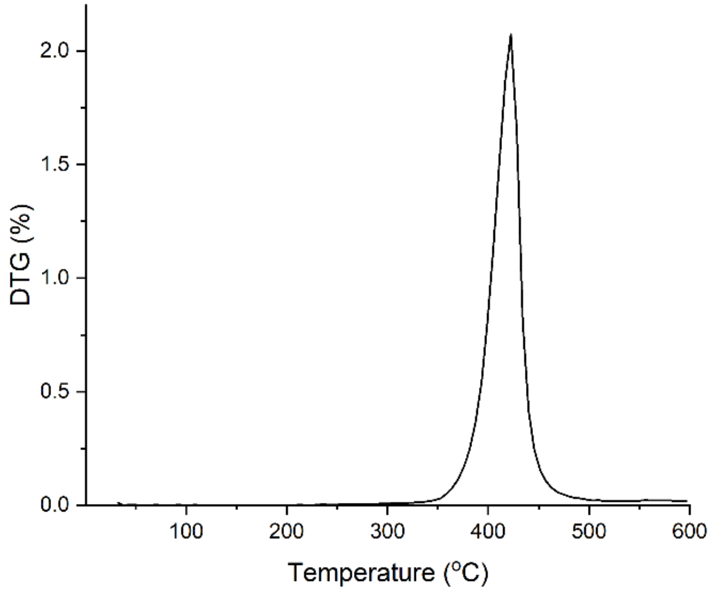

Figure S5: DTG curve of residual postconsumer waste textile after acid hydrolysis (43 wt% aq. HCl) for 3 h and measured under N_2 flow.

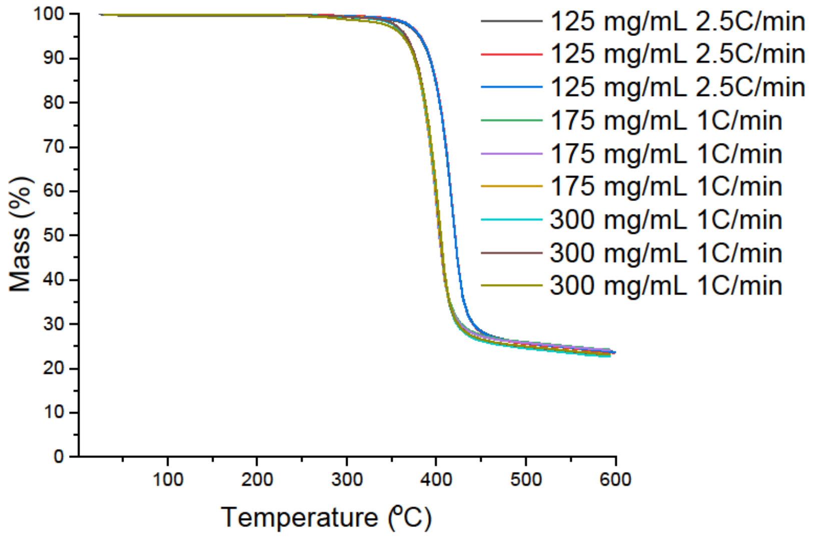

Figure S6: TGA of residual postconsumer waste textile after treatment with 43 wt% aq. HCl content of different textile loadings measured under N_2 flow. *Note: the difference in decomposition temperature is due to the use of different heating rates (2.5 and 1 °C/min).*

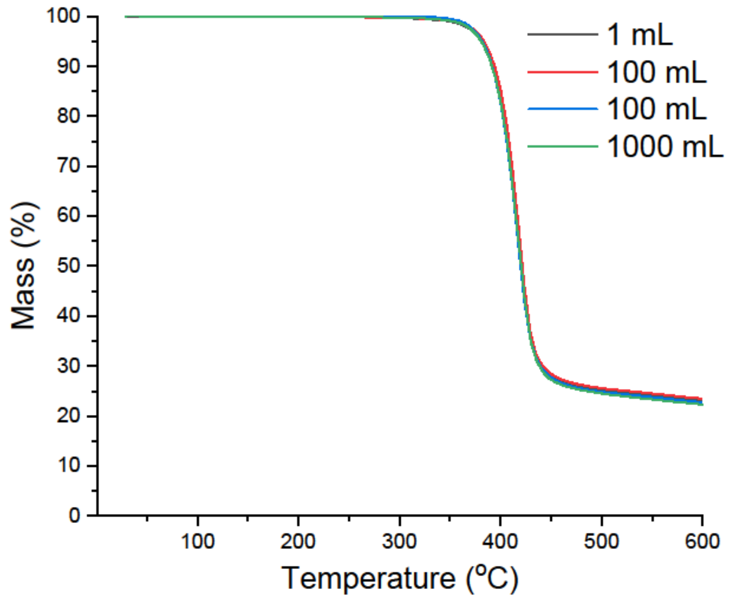

Figure S7: TGA of residual postconsumer waste textile after treatment with 43 wt% aq. HCl content on different scales measured under N_2 flow.

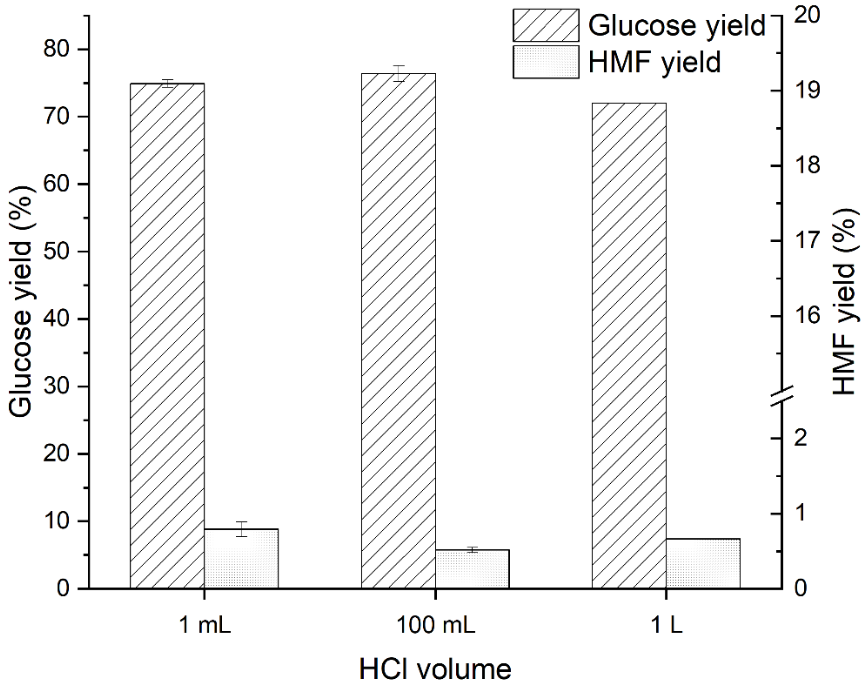

Figure S8: Effect of reaction volume on the glucose and HMF yield after hydrolysis (43 wt% aq. HCl, 1 d, static reaction) of the postconsumer waste textile. Shown values for 1 mL and 100 mL are averaged from three and two measurements, respectively, and the error bars indicate the standard deviation

Table S2: Pilot Plant run information.

	Material		Material in/output			Reaction parameters					
Run	Type	Cotton (%)	Mass textile [kg]	Mass HCl [kg]	Mass residual material [kg]	Filling rate [L/h]	Static reaction time [h]	Draining time [h]	T_{HCl} inlet [°C]	T_{HCl} outlet [°C]	Glucose yield [%]
1	Postconsumer waste textile	44	11.6	254	5.9	28	24	4	13	17	56
2	Pure cotton shirt	98	11.8	260	0.21	32	24	1	11.5	16.8	75
3	Mixed postconsumer waste textile	65	26.1	237	9.3	32	48	1	12.4	18.5	75

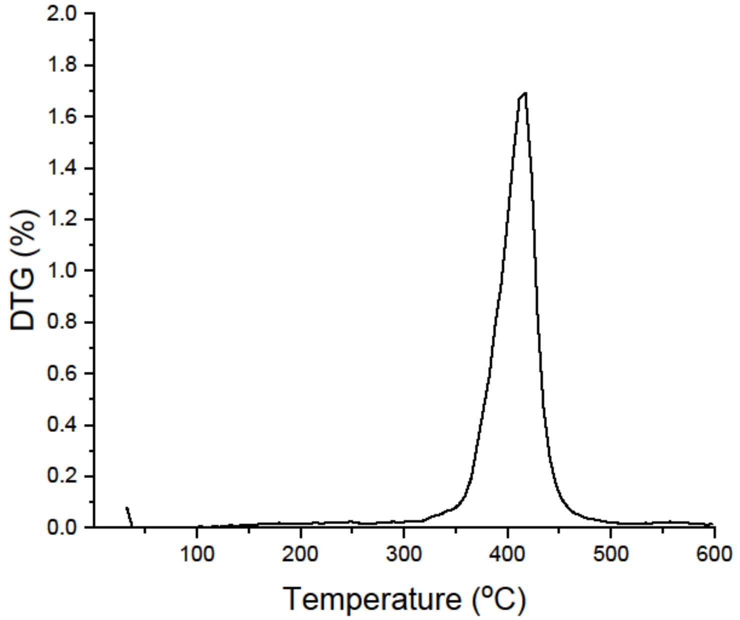

Figure S9: DTG curve of residual postconsumer waste textile after acid hydrolysis with 43 wt% aq. HCl at the pilot plant (run 1) measured under N_2 flow.

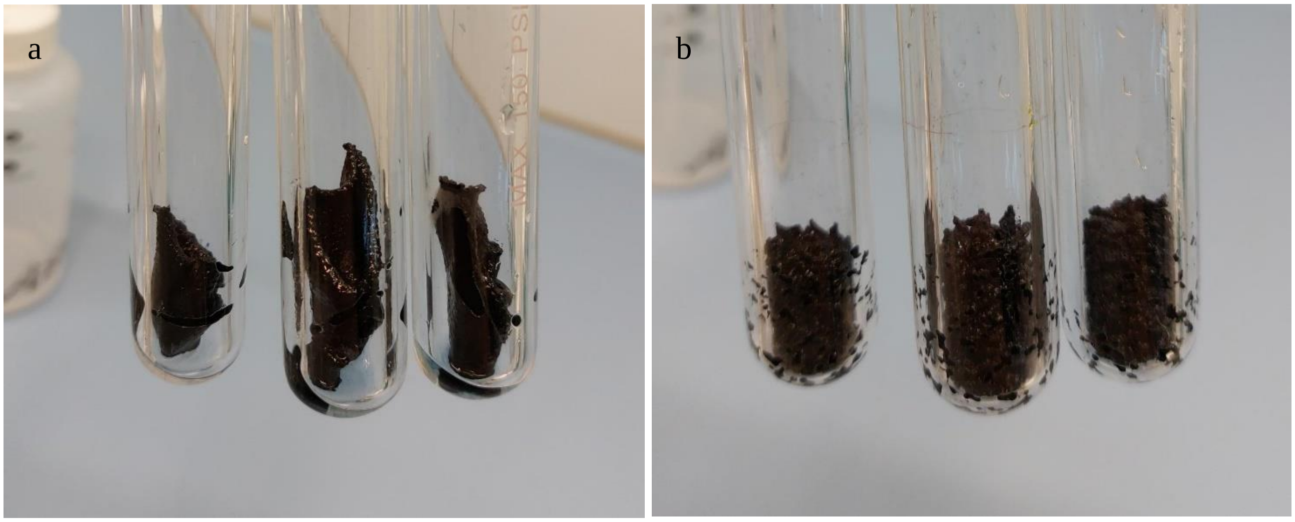

Figure S10: Images of textiles with normal (a) and increased (b) surface areas after acid hydrolysis (43 wt% aq. HCl, 1 day, 300 mg/mL)

Table S3: The effect of an increased surface area on the glucose and HMF yield of acid-hydrolyzed waste textile (43 wt% aq. HCl, 1 d).

		**Glucose yield**	**HMF yield**
50 mg/mL	Standard surface area	76% (SD 0.4%)	0.52% (SD 0.01%)
	Increased surface area	77% (SD 0.6%)	0.45% (SD 0.11%)
300 mg/mL	Standard surface area	39% (SD 0.5%)	0.29% (SD 0.01%)
	Increased surface area	42% (SD 0.1%)	0.28% (SD 0.03%)

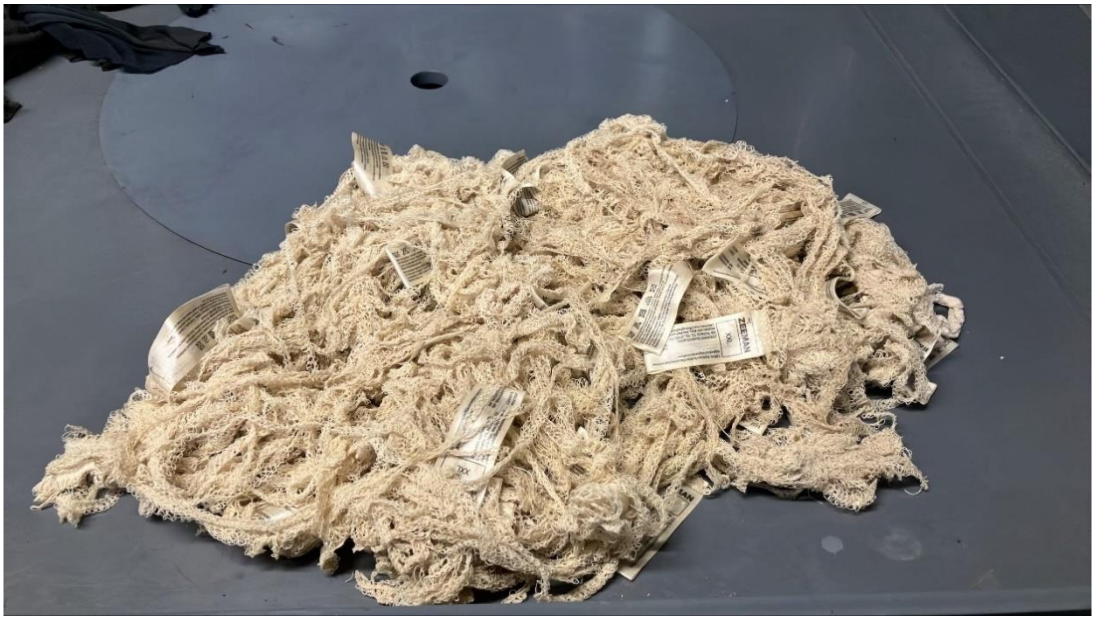

Figure S11: Polyester residue of pure cotton shirts after hydrolysis with 43 wt% aq. HCl in the DAWN pilot plant (run 2).

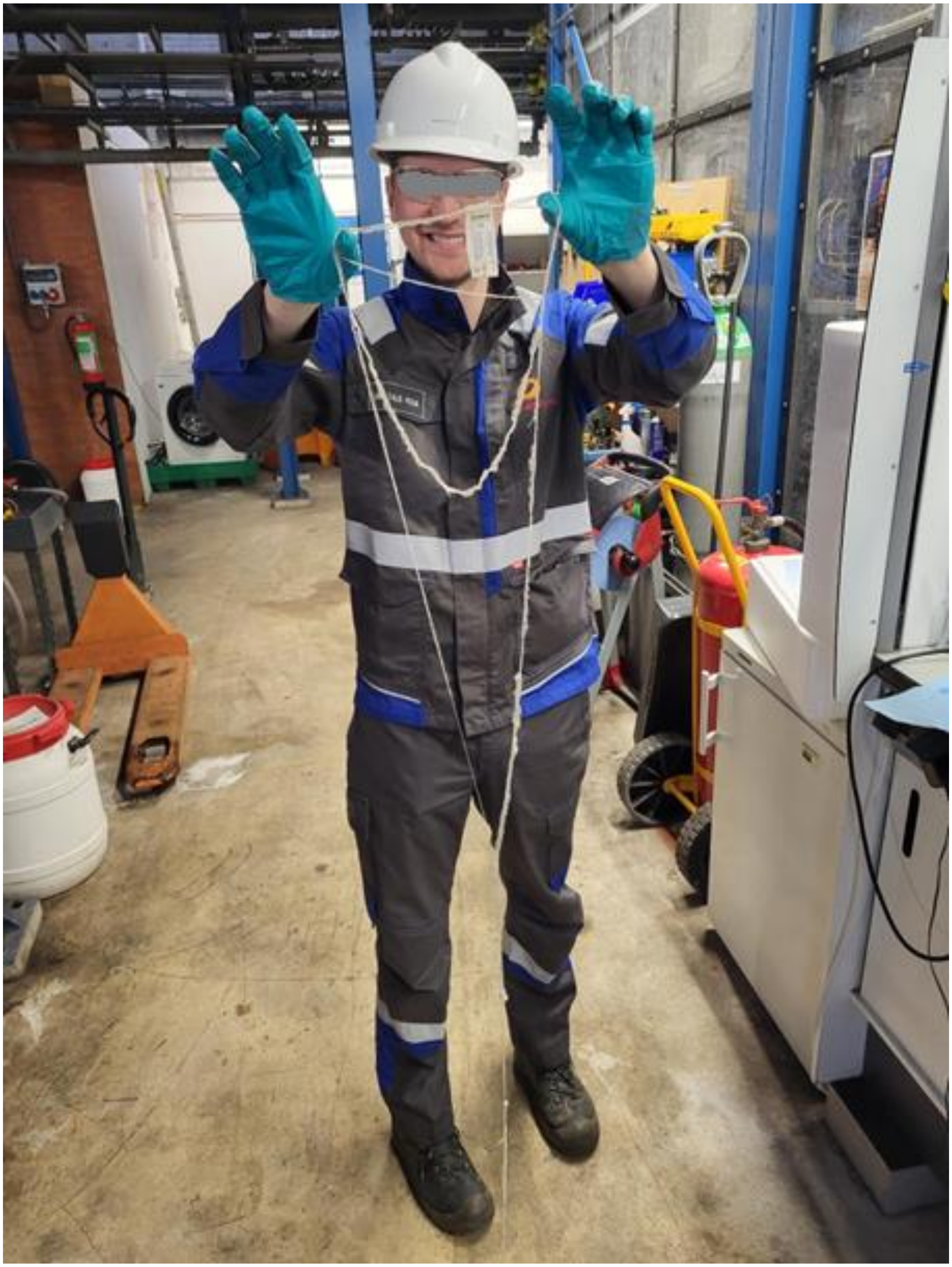

Figure S12: Polyester residue of one pure cotton shirt after hydrolysis with 43 wt% aq. HCl in the DAWN pilot plant (run 2).

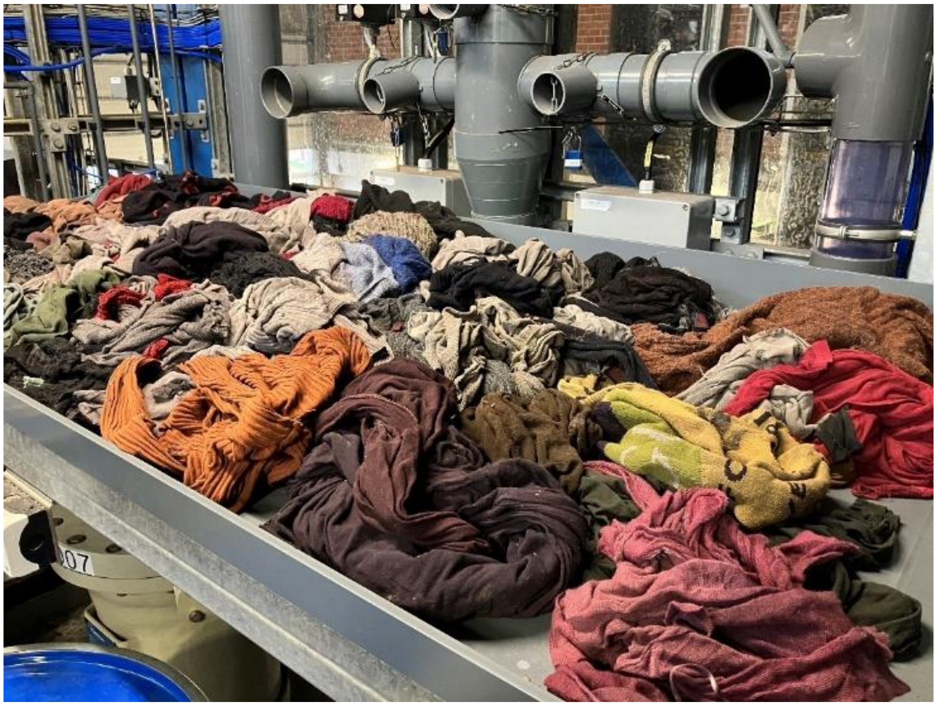

Figure S13: Polyester residue of postconsumer polycotton waste textiles after hydrolysis with 43 wt% aq. HCl in the DAWN pilot plant (run 3).

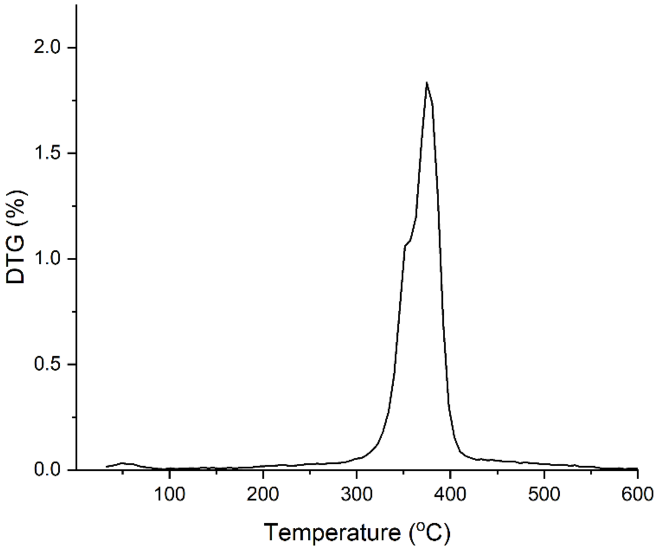

Figure S14: DTG curve of a residual postconsumer waste textile garment after acid hydrolysis with 43 wt% aq. HCl at the pilot plant (run 3) measured under N_2 flow. The shoulder in the polyester peak indicates the presence of cotton residue.

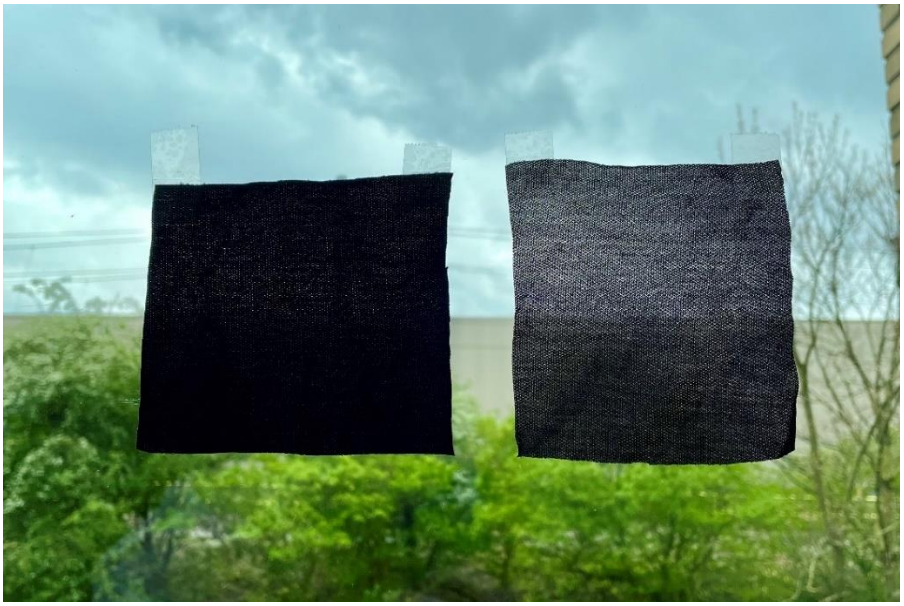

Figure S15: Light transmission through a polycotton waste textile before (left) and after (right) acid hydrolysis (43 wt% aq. HCl).

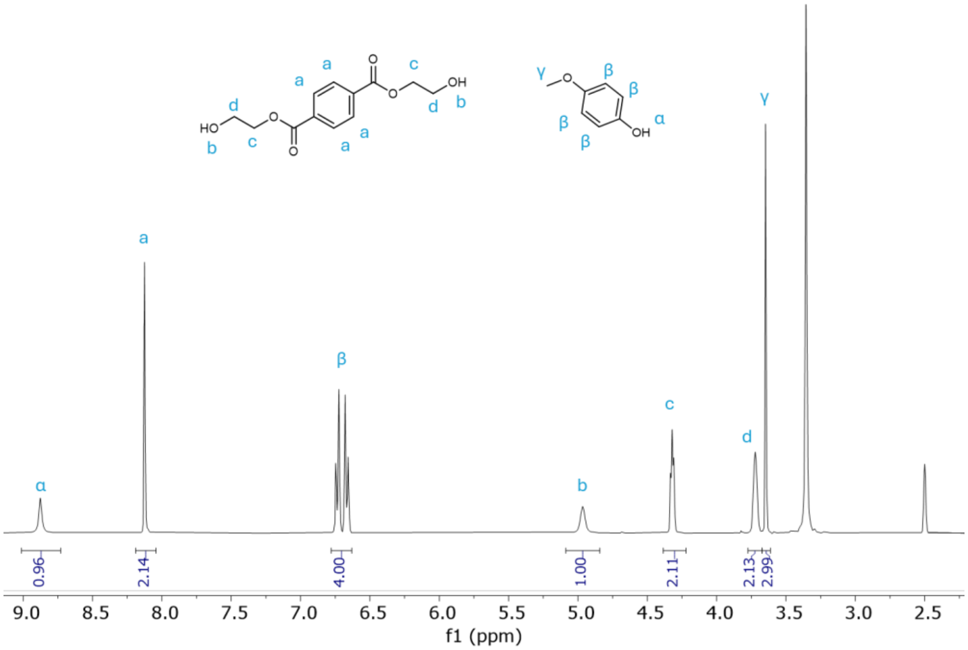

Figure S16: ^{1}H NMR spectrum of BHET obtained after glycolysis of residual PET from waste textiles. The protons labeled a, b, c and d are the protons corresponding to the protons in the aromatic ring (δH = 8.13 ppm, s, 4H), hydroxyl groups (δH = 4.96 ppm, t, 2H), methylenes ($-CH_2-$) adjacent to the -OH groups (δH = 3.71 ppm, m, 4H), and methylenes ($-CH_2-$) adjacent to the -COO groups (δH = 4.32 ppm, t, 4H), respectively. Additionally, 4-methoxyphenol is used to determine the purity of BHET. The protons labelled α, β, γ are the protons corresponding to the protons in the -OH group (δH = 8.88

ppm, s, 1H), the aromatic ring (δH = 6.72-6.68 ppm, m, 4H) and the methyl group (δH = 3.65 ppm, s, 3H), respectively. Sample was dissolved in DMSO-d_6.

SUMARY CONCEPTUAL PROCESS DESIGN (CPD) AND TECHNO-ECONOMIC ANALYSIS (TEA)

AVANTIUM YUKON POLYCOTTON PROCESS.

Process Design Center, Breda, The Netherlands (https://www.process-design-center.com)

May-July 2024

BASIS OF DESIGN

This description summarizes the results of the Conceptual Process Design (CPD) and techno-economic assessment (TEA) of converting polycotton textile waste into 5-(chloromethyl)furfural (CMF) and subsequently 5-(methoxymethyl)furfural (MMF), with residual polyethylene terephthalate (PET) as a valuable co-product.

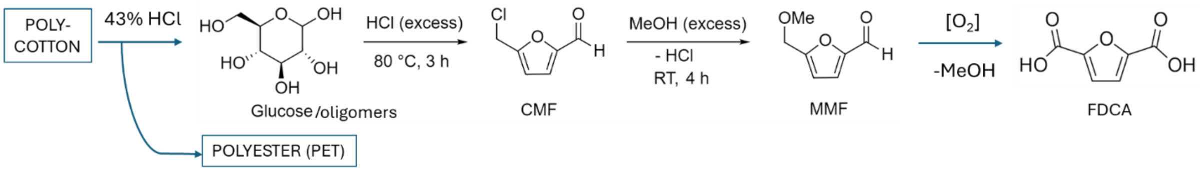

The process consists of the following main steps:

- A battery of hydrolysis reactors where textile is contacted with 43wt% hydrochloric acid solution in a simulated moving bed reactor operation. This results in the production of hydrolysate containing oligomeric and monomeric glucose.
- The (oligomeric) glucose-hydrolysate stream is phase separated to remove the humins fractions and PET debris, and then sent to the CMF reactors. The solid residual (PET) is unloaded from the hydrolysis reactors, pressed, dried, neutralized, washed and dried again. HCl released upon drying is recovered and sent to the HCl reconcentration section.
- In the CMF section, hydrolysate with HCl is converted into CMF in two counter-current extractive CSTRs in series, using chlorobenzene as extraction solvent. The liquid is phase separated to obtain the organic fraction containing CMF. The chlorobenzene is evaporated to recover the solvent. The HCl-containing aqueous phase is sent to the HCl reconcentration section.
- In the MMF section, CMF and methanol are reacted to form MMF at room temperature and atmospheric pressure, with an in-situ neutralization operation. The outflow stream of this reaction is sent to a column to recover methanol, followed by MMF extraction and workup by distillation.
- The HCl reconcentration section consists of a dual-pressure distillation section to circumvent the HCl/water azeotrope and an absorption section to produce 43% HCl solution for reuse in the hydrolysis reactor section.

The economic evaluation was conducted on a February 2024, Delfzijl (Netherlands) and Euro basis for a 40 kt/yr dry waste textile feed capacity, assuming 8,000 operating hours per year. On this basis the process has an MMF production capacity of 11 kt/yr. The estimated total capital investment amounts to a fixed Capital Investment (FCI) of € 103M (a total Capital Investment (TCI) of € 116M).

The most expensive process sections are CMF production from hydrolysate, textile hydrolysis, and solid (PET and humins) handling. Further investments are potentially required for unresolved technical issues. Based on a dry waste textile price of 100 €/t, a PET co-product price of 1000 €/t, a depreciation time of 15 years, and exclusion of financing cost, the estimated production cost per metric ton of MMF is:

Raw materials	€ 697
Power and utilities	€ 361
Wastewater treatment	€ 243
Labor and supervision	€ 272
Maintenance and repairs	€ 469
Other direct cost	€ 106
Indirect cost	€ 727
General expenses	€ 180
Depreciation	€ 626
Co-products	€ -1,822
MMF manufacturing cost	€ 1,859

A sensitivity study was conducted, showing that particularly the plant capacity, the polyester co-product value and maintenance and repairs (indirect indication of CAPEX) have a major impact on the MMF production cost.

Table summarizing the investigated range of variables and their effect on the MMF production cost. Utility average cost represents the weighted average of fuel, steam and electricity cost.

Parameter	Unit	Base value	Worst case	Best case
Plant capacity	*kt/a*	40	10	100
Polyester price	€/ton	1000	500	1300
Maintenance	of FCI	5%	10%	2%
Textile price	€/ton	100	200	0
Depreciation time	yr	15	10	20
Utility avg. cost	€/GJ	8.9	13.29	6.20
Solvent price	€/ton	1000	2000	500

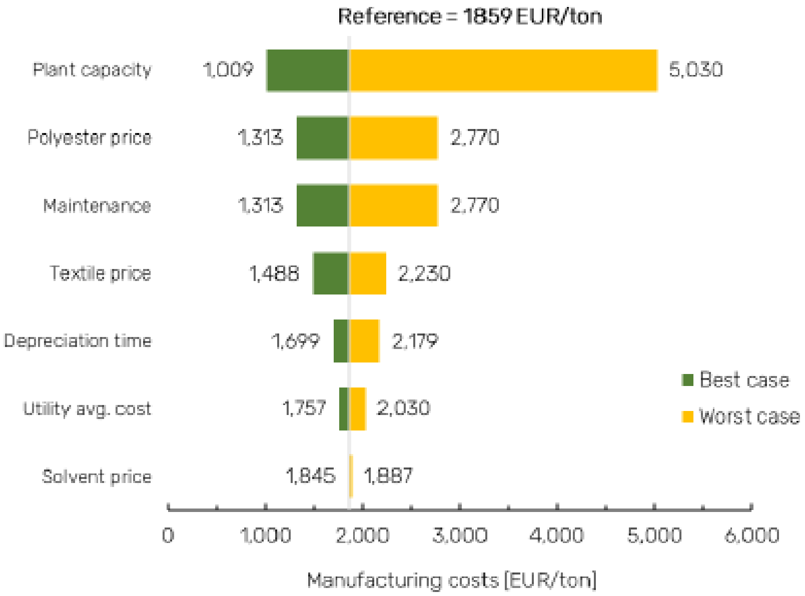

The Tornado chart shows the impact of variables (see Table above) on the MMF production cost.

The technology applied for textile feedstock appears a technically and economically interesting alternative for hardwood as feedstock. The main benefits are the one-stage hydrolysis with one concentration of HCl, the avoidance of the hemicellulose value chain, and the positive economics also at reduced MMF production capacity. Main downside is the lower hydrolysis reactor productivity due to the lower feedstock bulk density.

Feedstock

The main feedstock is textile with a composition of 50% cotton and 50% polyester (mass). It is pretreated (removal of zippers and button etc.) and shredded before use, resulting in a bulk density of 108 kg/m^3.
The HCl concentration to the reactor and for makeup is 43% and 30% (mass), respectively.
Natural gas is imported (OSBL) with a pressure assumed suitable for the burner. A typical inlet pressure for compressed air for the burner is applied.
Solid NaOH is dissolved in water before use. $Ca(OH)_2$ is mixed with water to make a paste to facilitate the feeding systems. Further dilution is executed where necessary.
Chlorobenzene and methanol are stored onsite in storage vessels.

Physical properties

The electrolyte NRTL model (ELECNRTL), based on the NRTL liquid activity coefficient model, and the Redlich-Kwong equation of state model (EoS) for the gas phase have been used for simulation in Aspen Plus, version 12.The EHCLFF data package was used for Henry components.
For HCl and methanol, data seem to suggest an azeotrope, but the physical method ELECNRTL does not describe an azeotrope. This is, however, not such an issue as the HCl in the CMF section is neutralized.

Additional relevant components were added. In the absence of physical properties, their physical properties have been estimated using group contribution methods. The (hemi-)acetals of MMF and CMF have not been considered as separate components.

Basis of design

The plant capacity is 40 kt/yr waste textile shred on dry basis, resulting in an MMF production capacity of 11 kt/yr. The location or site is Delfzijl, The Netherlands. The conceptual process design has been made based on the operating and performance data and information described in this paper. Below, the main process design data for the major unit operations are summarized.

Hydrolysis

The design is simplified (versus the wood-based process) to a single stage (main) hydrolysis concept, which operates the reactor in simulated moving bed mode. In total, the hydrolysis reactor system comprises of 5 reactor vessels, each of 360 m^3, which rotate in cycles of 5 steps, each of 8 hours:

- Loading/unloading 8 hours
- Reaction 3 x 8 hours = 24 hours
- Washing 8 hours

The steps are described below in more detail.

1. Hydrolysis (R1/R2/R3)

The loaded and flood-filled reactor (R5) is transformed to position R1 and operated for 24 hours in countercurrent mode (R1, R2, R3) with the highly concentrated HCl coming from the R4 washing stage. The input data is described below.

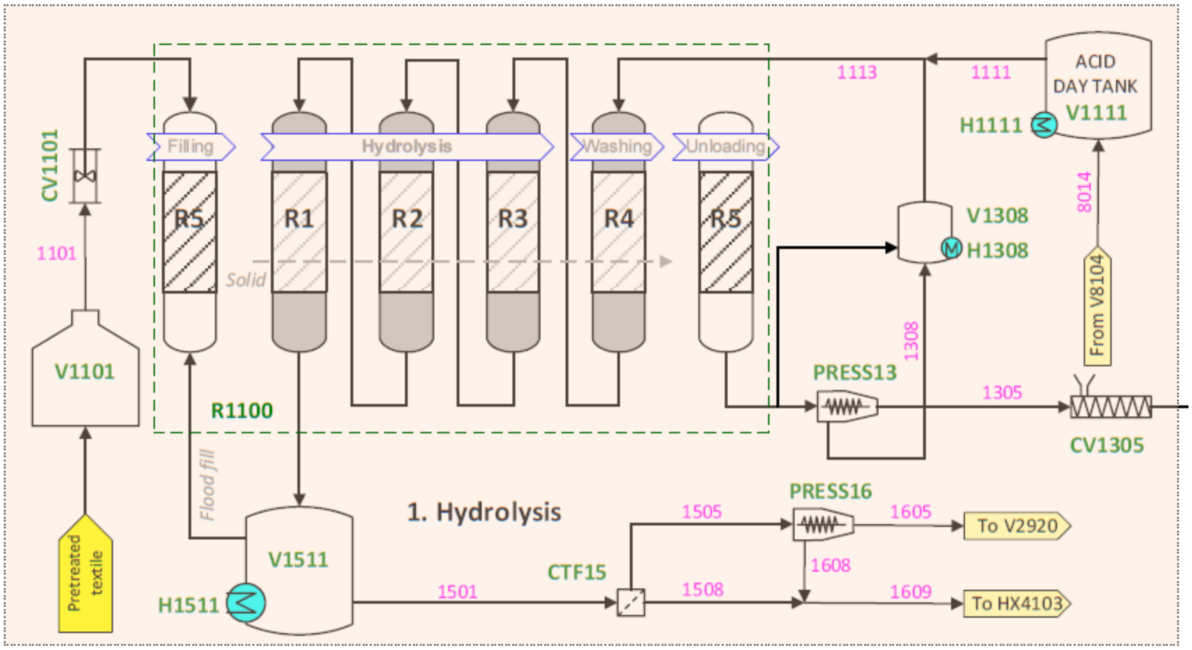

Process input	Hydrolysis
HCl concentration (feed)	43%
Feed ratio fresh/total HCl solution to dry textile	8 g/g
Contact time	3*8 hr

The HCl flowrate is adjusted to achieve the indicated feed ration of 8 kg/kg. The resulting hydrolysates concentration is 5.7%, in line with the experimental results.

2. Washing (R4)

After three reaction stages (R1/R2/R3) the reactor is transformed to the R4 washing stage where fresh and recycled 43% HCl is fed from acid day tank V111 and recycle tank V1308. This stage is used to wash the residual hydrolysate/sugars from the cotton depleted textile (8 hours).

3. Loading/unloading (R5)

After washing (R4), the reactor is transformed to the unloading position (R5). First the liquids are drained from the reactor and collected in V1308. Then the residual textile/polyester is unloaded by opening the larger reactor valve, and sent to a screw press (Press13).

Next the reactor is loaded with fresh textile from storage vessel V1101 and flood filled with hydrolysate product liquid, which is collected in V1511 (8 hours).

The hydrolysis performance is following:

Conversion

- 0% of polyester is converted
- 100% of cotton is hydrolyzed to a mixture of glucose monomers and oligomers
    - in 80% yield to hydrolysate (65% glucose, 14.5% oligomer (DP2), 0.5% HMF)
    - 20% to humins (in model as "$C_{23}H_{28}O_{13}$").

Recovery

- 1% of polyester effluents from the reactor with the aqueous hydrolysate stream
- Humins
    - No differentiation between High Density & Low Density humins
    - Assuming same behavior as other soluble solutes
- Liquid carryover: solid polyester = 2.88 (mass) after draining
- Concentration of hydrolysates in the carried liquid = 10% * Concentration of hydrolysates in the liquid effluent (Assumption)

Reactor design

The hydrolysis reactor design considers fiber reinforced plastic reactors of 360 m^3 according to the Plasticon quotation.

- Operating temperature: 15°C
- Design pressure: 5 bar
- Capacity: 40 metric tons of textile

Given the low bulk density of the textile feedstock of 108 kg/m^3, a reactor vessel is loaded with 40 tons of textile and 333 m^3 of hydrolysate flood filled from tank V1511.

PET/humins processing

PET press (PRESS 23 & 25)

- Operating temperature: 15°C
- Pressure: atmospheric
- PET product moisture (H_2O+HCl) content: 50%
- Max. capacity: 2.5 t/h PET (dry basis)

PET dryer (Dryer 21)

- Co-current contact
- Operating temperature: hot flue gas 200 → 56.9 °C solid 20→56.9 °C
- Pressure: atmospheric
- Max. capacity: 2.5 t/h PET (dry basis)
- PET product moisture content: 1% (assumed)
- PET product HCl content: 1% (assumed)

PET neutralization and washing

- Belt filter
- Water: PET (dry) = 2.37 (mass)

To reach the saturation point; extra free liquid due to the liquid carryover

- NaOH: stoichiometric amount

Solution prepared from the liquid effluent of water washing; no further specification

- Product temperature: 20 °C
- Pressure: atmospheric
- Max capacity: 2.5 t/h PET (dry basis)

PET dryer 2 (Dryer 26)

- Co-current contact
- Operating temperature: hot flue gas 200 → 49.8 °C; solid 20→49.8 °C
- Pressure: atmospheric
- Max. capacity: 2.5 t/h PET (dry basis)
- PET product moisture content: 1% (assumed)
- PET product HCl content: 0% (assumed)

Humins neutralization and pressing

- $Ca(OH)_2$: first mixed with water to form paste; diluted further to sufficiently wet humins
- Neutralized in a stirred tank
- Pressed in a screw presser
- Moisture content of pressed humins: 50%
- Temperature: 20 °C

- Pressure: atmospheric

<u>HCl/H_2O evaporation</u>

The design of this section was performed by SGL Carbon in 2020 for the wood CPD.
The design of SGL covers the following elements:

- Polyester Dryer Gas Treatment (PDGT) – Section 3
- Dual pressure distillation (DPD) – Section 6
- HCl absorption (HPA) – Section 8

SGL equipment has been scaled to accommodate the changes in the mass and energy balance. A scaling exponent factor of 0.7 was applied.

<u>Hydrolysate to CMF</u>

The CMF reactors operate as extractive reactors converting hydrolysate while extracting the products with chlorobenzene. The conditions have been determined by batch laboratory reactions. The selected operating conditions and performance of the CMF reactor are:

- Temperature: 80 °C
- Residence time (batch): 3 h
- Pressure: 10 bar (to keep solution in liquid phase)
- Chlorobenzene / hydrolysate ratio: 1 kg/kg
- C6 sugar conversion: 100 %
- To CMF: 90 %
- To Humins: 9.5 %
- To levulinic acid and formic acid: 0.5 %

Since the reactors are operated in a countercurrent continuous (CSTR) mode rather than in batch mode, it is considered that two CSTRs in series, each with a residence time of 1.5 h are sufficient to ensure full conversion. Assuming first order kinetics, approximately 90% (oligomeric) glucose conversion is reached in the first reactor and the remainder in the second reactor.

CMF is converted to MMF using methanol as reactant at the following conditions:

- Temperature: 25 °C (ambient conditions)
- Residence time (batch): 3 h
- Temperature: 25 °C (ambient conditions)
- Residence time (batch): 3 h
- Pressure: 1.2 bar
- Methanol/CMF ratio: 3 kg/kg
- CMF conversion: 100%
- CMF acetal yield: 5%
- MMF acetal yield: 95%

Utilities

With the exception of coolant, all utility generation is considered outside battery limits (OSBL). The following utilities are imported from over the fence:

- IP steam (29 bar, 255 °C)
- LP steam (3.9 bar, 180 °C)
- Natural gas
- Cooling water (27 °C)
- Electricity

Coolant is generated by a dedicated refrigeration unit (SE1900), which generates coolant at a temperature of below 5 °C at a calculated coefficient of performance (COP) of 5.95. Coolant is used in the following units: H1111, H1307, H1308, H5102, H6311, E8010.

Economics

The economic evaluation is performed on the basis of the capital investment and the total production cost. General parameters of the cost estimation procedure are:

- Cost date: February 2024
- Currency: Euro
- Location: NL (Delfzijl)
- On-stream time: 8,000 h/year

Cost estimates were determined based on (capacity scaled) vendor quotations and for the remainder based on the conceptual process design (CPD) using PDC's proprietary cost estimation tool PROSYN® Costing. PDC's cost estimation tool covers main equipment and enable a quick design and capital estimate of process equipment on the basis of key equipment parameters, providing a capital and production cost estimate that is considered fit-for-purpose for comparison of conceptual design alternatives. The costing estimation tool is based on the method of Ulrich [Ulrich, 2004].

Capital investment

The Bare Module Capital (BMC) for each listed equipment item is determined or taken from the vendor quotations.

For cost estimation the BMC includes the following elements:

- Direct cost Purchased Equipment Cost (PEC), also referred to as Free-On-Board (FOB) cost
- Installation materials, which covers piping, concrete (foundation), steel (structural support), instruments, electrical materials, insulation, and paint.
- Direct labor for installation, which covers the wages of laborers who install the equipment
- Indirect cost Freight, insurance and taxes
- Construction overhead
- Contractor engineering expenses

The sum of the BMC of all listed equipment items is entered in the capital cost estimation sheet. An allowance is included to cover unlisted equipment, amounting to 15% of the Subtotal BMC excluding vendor quoted equipment, for which an unlisted equipment allowance of 5% is used, resulting in an overall unlisted equipment factor of 11% of the Subtotal BMC. Examples of unlisted equipment are vacuum units, pumps, additional vessels, etc.

Quoted equipment scaling of the cost data was performed using an exponent of 0.7 based on the duties or throughput. To arrive at the BMC for vendor quoted equipment installation factors were calculated

on the basis of the following data:
Installation factors [Guthrie, 1974]:

- Piping 0.45
- Civil 0.05
- Steel 0.03
- I&C 0.10
- Electrical 0.02
- Insulation 0.05
- Paint 0.01
- Labour (Cp + Cm) 0.37

Piping factors [Ulrich, 2004]

- Crushers, mills, grinders 0.03
- Furnaces 0.15
- Process vessels (vertical) 0.60
- Pumps 0.30
- Separators 0.25

Indirect costs [Guthrie, 1974]

- Freight 0.08
- Construction overhead 0.70
- Contractor engineering 0.15

This resulted in the calculated installation factors indicated in Table 2.1.

Table 2.1: Calculated installation factors for quoted equipment

Supplier	Quoted equipment	Installation factor (calc)	Remarks
Andritz	Wood yard	1.05	Includes freight, mechanical and electrical installation, start-up & commissioning, spare parts. Civil scope is excluded
Andritz	Wood yard WWTP	1.05	Includes freight, mechanical and electrical installation, start-up & commissioning, spare parts. Civil scope is excluded
Andritz	Polyester un-loading valve	2.05	Mechanical & Electrical erection, civil scope, utility piping connection and large part of electrical (MCC's, power supply, switchboard, cabling) and indirect costs are excluded.
Plasticon	Hydrolysis reactors	2.52	Only reactors, labor costs adjusted
Klinger	Polyester un-loading valve	1.00	Only valves, installation in reactor scope
Sulzer	Polyester un-loading pump	2.03	Only pumps
Valmet	Screw press polyester	1.72	Only screw press, labor costs adjusted for expensive materials
Feeco	Rotary dryer polyester	2.51	Only rotary dryer
CPM	pelletization unit	1.99	Installation, civil, cabling, indirect costs are excluded.
GEA	Disk stack centrifuges	1.44	Installation, civil are excluded
SGL	HCl evaporation and recovery	2.1	Based on factors provided by SGL

To obtain the **Total Module Capital Cost (TMC)** the following cost items are added up to the Total Bare Module Capital (TBMC):

- Contingencies: set to 30% of the TBMC. This contingency value is selected because of the first-of-its-kind project.
- Engineering: set to 10% of the TBMC.
- Construction: set to 10% of the TBMC.
- Automation and control: set to 8% of the carbon steel equivalent TBMC.
- Fee (for contractor): 6% of the TBMC.

The carbon steel equivalent TCBM for vendor quoted equipment is calculated by bare module factor ratios of carbon steel versus actual material of construction.
The **Total Grassroots Capital Cost**, which may be considered equivalent to the **Fixed Capital Investment (FCI)** is the Total Module Capital (TMC) plus the cost for site development, auxiliary buildings and offsite facilities. No cost for land or land lease has been included.

- Site developments, 2% of TMC
- Auxiliary building, 4% of TMC
- Off-site facilities, 20% of TMC

According to Guthrie [Guthrie,1974] auxiliary buildings include control rooms, product warehouses, laboratory, administrative and office, medical, restaurant/cafeteria, garage, guard and safety, personnel building, maintenance shops and building services (plumbing, heating, ventilation, air conditioning, lightning, elevators, intercommunication services, painting, etc.).

Off-site facilities cover:
- Utilities: steam, water, power, refridgeration, compressed air, waste disposal, etc.
- Facilities: wells, river water intake, water treatment, boilers, cooling towers, water storage, electric substations, refrigeration and air plans, fuel storage, fire protection, etc.
- Non-process equipment: office furniture and equipment, lab equipment, automotive and garage equipment, medical equipment, locker-room equipment, fire extinguishers, hoses, fire engines, etc.
- Distribution and packaging: raw-material and product storage and handling equipment, product packaging equipment, blending facilities, loading stations, etc.

The **Total Capital Investment (TCI)** is the sum of the following items:
- Fixed Capital Investment (FCI)
- Working Capital, 10% of FCI
- Start-up Expenses, 2% of FCI

Production cost
The total production costs are calculated as the sum of the following elements:
- Direct production costs
    - Raw-materials and supplies
        - Waste textile 100 €/t (dry basis)
        - Methanol 410 €/t
        - Chlorobenzene 1000 €/t
        - Calcium hydroxide 150 €/t (dry)
        - Sodium hydroxide 233 €/t (dry)
        - HCl (30%) 82.5 €/t (equivalent to 275 €/t for pure HCl (100%)).
        - Process water 0.86 €/t
    - Co-Products and waste streams
        - PET 1000 €/t
        - Wet/dry humins no value/cost assumed
        - Heavy/light-ends no value/cost assumed
        - Wastewater 1.50 €/t fixed / 1.05 €/t variable + COD cost
          COD = 40 €/PU, PU = V[m^3/y]/1000*COD[mg/L]/49.6
        - HCl (30%) 82.5 €/t (equivalent to 275 €/t for pure HCl (100%)).
    - Operating labor: Average wage Delfzijl plant operators = 52,000 €/yr, 5 shifts.
    - Supervision (15% of Operating labor)
    - Power and utilities:
        - LP steam 10.8 €/t (variable) + 6.25 €/t (fixed price = 50 k€/yr per ton/hr steam capacity).
        - IP steam 14.78 €/t (variable) + 6.25 €/t (fixed)
        - Natural gas 20 €/MWh (HHV) = 6.2 €/GJ (LHV)
        - CW 0.04 €/m^3
        - Electricity 60 €/MWh

Maintenance and repairs (5% of grass roots capital)

- Operating supplies (15% of Maintenance and repairs)
- Laboratory charges (15% of Operating labor).

No cost or credits for patents and royalties and auxiliary materials have been considered.

- Indirect costs
    - Plant overhead costs (60% of Operating labor, Operating supervision and Maintenance and repairs)
    - Local taxes (2% of grass roots capital)
    - Insurance (1% of grass roots capital)
- General expenses
    - Administrative costs (25% of operating labour)
    - Distribution and marketing costs (1% of Manufacturing Expenses)
    - Research and development (1% of Manufacturing Expenses)
- Depreciation (6.7% of grass roots capital = 15 years depreciation time)
- Financing cost is excluded

The MMF manufacturing or production cost in €/t is calculated by dividing the total annual production cost by the annual MMF production.

References for CPD and TEA:

[Ulrich, 2004] G.D. Ulrich & P.T Vasudevan, Chemical Engineering - Process Design and Economics, A Practical Guide, 2nd Ed., Process Publishing, Durham New Hampshire, 2004.

[Guthrie, 1974] Guthrie, K.M., "Process Plant Estimating Evaluation and Control", Craftsman Book Company, 1974.

References

1. Loo, S.-L., Yu, E. & Hu, X. Tackling critical challenges in textile circularity: A review on strategies for recycling cellulose and polyester from blended fabrics. *J Environ Chem Eng* **11**, 110482–110508 (2023).
2. R. Speight, R. Graham, I. O'hara, J. Zhanying Zhang, D. Moller and A. Jones, "A system and process for the separation and recycling of blended polyester and cotton textiles for re-use". Australia Patent WO2020252523A1, 24 December 2020.
3. F. G. Barla, T. Showalter, H.-C. Su, J. Jones and I. Bobe, "Methods for recycling cotton and polyester fibers from waste textiles". United States 18 July 2019.
4. S. Flynn and C. Stanev, "Methods and systems for processing mixed textile feedstock, isolating constituent molecules, and regenerating cellulosic and polyester fibers". United States Patent US11034817B2, 15 June 2021.
5. E. Yee Man Keh, L. Yao, X. Liao, Y. Liu, K. Cheuk and A. Chan, "Method for separating and recycling a waste polyester-cotton textile by means of a hydrothermal reaction catalyzed by an organic acid". United States Patent US11396114B2, 26 July 2022.
6. A. Harlin, J. Mäkelä and S. Siren, "Separation of polycotton blends". Finland Patent FI130401B, 14 August 2023.
7. K. Valta and E. Sivonen, "Method for manufacturing cellulose carbamate". Finland Patent WO2003064476A1, 7 August 2003.
8. Haslinger, S., Hummel, M., Anghelescu-Hakala, A., Määttänen, M. & Sixta, H. Upcycling of cotton polyester blended textile waste to new man-made cellulose fibers. Waste Management 97, 88–96 (2019).
9. R. Herchl, C. Klaus-Nietrost, S. Theis and C. Weilach, "Process for recovering starting materials from mixed textile wastes". Austria Patent WO2021115931A1, 17 June 2021.
10. M. Lindström, C. Lindgren and G. Hendriksson, "Cellulose fibers". Sweden Patent WO2018104330A1, 14 June 2018.
11. G. J. Brinks, G. H. Bouwhuis, P. B. Agrawal, H. Gooijer and J. J. Oelerich, "Method for producing regenerated cellulose fibers from cotton containing textile waste". The Netherlands Patent WO2017135816A1, 10 August 2017.
12. J. Bogren, J. Parkas, G. Schild and A. Borgards, "New process and a dissolving pulp manufactured by the process". Sweden Patent WO2013178608A1, 5 December 2013.
13. H. Brelid and J. Bogren, "A process for separation of the cellulosic part from a polyester and cellulose composition". Sweden Patent WO2020013755A1, 16 January 2020.
14. A. Walker, J. E. S. Reid and L. Hauru, "Recycling process". Great Britain Patent WO2020221932A1, 5 November 2020.
15. Dee, S. J. & Bell, A. T. A study of the acid-catalyzed hydrolysis of cellulose dissolved in ionic liquids and the factors influencing the dehydration of glucose and the formation of humins. *ChemSusChem* **4**, 1166–1173 (2011).
16. Rasmussen, H., Sørensen, H. R. & Meyer, A. S. Formation of degradation compounds from lignocellulosic biomass in the biorefinery: sugar reaction mechanisms. *Carbohydr Res* **385**, 45–57 (2014).
17. Yang, G., Pidko, E. A. & Hensen, E. J. M. Mechanism of Brønsted acid-catalyzed conversion of carbohydrates. *J Catal* **295**, 122–132 (2012).
